# A Case of Tympanites, in an Infant, Relieved by the Operation of the Paracentesis

**Published:** 1802-03

**Authors:** W. Coley

**Affiliations:** Surgeon, in Bridgenorth


### *A Case of Tympanites, in an Infant, relieved by the Operation of the Paracentesis.*

*With Remarks on the Case; and a critical Analysis of the Sentiments of the principal Authors who have written on the Disease. To which is subjoined, an Account of the Operation of the Acupuncture, as practised by the Japanese in Diseases analogous to the Tympany. Communicated to the Editors by,* Mr. W. Coley, *Surgeon, in Bridgenorth.*

B. A male infant aged eight months, was brought to me on April 29, 1797. On inquiry and examination I found him to have a moſt prodigiouſly diſtended abdomen, the diſtenſion extending quite to the thorax, the whole of which appeared to be greatly protruded. The ſwelling was equal over every part; and the whole of the integuments, from the ſternum to the pubis, as well as laterally, ſo far as diſtenſion could go, was of that extreme fulneſs that a bladder, however tightly filled, could not be more tenſe, or leſs elaſtic, for it would not admit of the ſmalleſt compreſſion. A ſonorous, or ſounding noiſe, on tapping the part with the hands was very clearly perceptible; and I thought I diſcerned an obſcure fluctuation in the hypogaſtrium, immediately after a diſcharge of urine from the bladder, which fortunately happened to take place at this time. The limbs were preternaturally ſmall; and the weight of the child evidently leſs than it could poſſibly have been, had the ſwelling conſiſted of an accumulation of any watery fluid. Its countenance was ſhrivelled and ſickly; there were a pallor and coldneſs of the ſkin, dyſpnœa, a weak pulſe, and a general muſcular emaciation. With theſe ſymptoms I alſo found that it had lately nauſeated the breaſt, by which it had been hitherto almoſt entirely ſuſtained; that it was frequently pining, had but little ſleep, was apparently in much pain, and had for ſeveral days loſt the power of relieving itſelf by inteſtinal crepitation. He paſſed from one to three looſe natural ſtools daily, and had ſo done from the time of his birth; and the urine was evacuated in proper quantity. The girth of the body, carefully meaſured over the moſt protruding part of the abdomen, was twenty-four inches and a half.

The origin of this diſeaſe was coeval with the birth of the child, for at that period the abdomen was obſerved to be tenſe and fuller than natural. For ſome months the progreſs of the ſwelling was ſlow, but evidently increaſing and continuing tenſe. Until very lately he had often paſſed wind downwards from his bowels with great force, by which he was generally relieved.

No

No remedies were made ufe of, or any particular notice taken of this intumefcence until a few weeks before I faw him; when, on account of his being in other refpects ill, and the fwelling beginning rapidly to increafe, he was taken to a medical gentleman who gave him fome purging pills; from which however no benefit was obtained.

I directed ʒjſs of the following ointment to be tenderly, but thoroughly rubbed into the abdomen every night; and one of the diuretic powders to be taken twice a day.

℞. *Ung. digital.* ℥*vj. Ung. hyd. fort. Sap. moll. a.* ʒ*jſs. M.*

℞. *Fol. digit. pulv. gr. iij. Sacch. alb.* ʒ*j m. f. pulv. xii.*

May 5. The child living in the country, I had not feen or heard from him until this day, when he was again brought to me. On the moft fuperficial view of his countenance, it was eafy to difcern that fome material amendment had occurred. The eyes, that before were inirritable and hardly moved in the fockets, were now clear, active, and noticing the different objects around; while his cheeks glowed with natural rednefs, and were plumped with increafing flefh. The appetite was voracious; his mother obferving that it was as much as fhe could do to fupply him with food and the milk of her breafts; and that he ate bread and butter greedily, though he had never taken any before. The fwelling had decreafed two inches, evidently the effect of a diuretic operation from the medicines. On examining the abdomen, which was done while he was held upright, it felt evidently lefs tenfe, more elaftic, (becaufe admitting of refiftance) ftill fonorous, but with a fluctuation, as I thought, manifeftly perceptible in the hypogaftrium. The thorax as well as the abdomen appeared lefs diftended.

On the fecond night after taking and ufing the medicines, it was obferved that he made an unufual large quantity of urine; and from that time to the prefent he continued paffing large quantities in the day, and wetting feveral cloths in the nights. His mother hardly recollects which occurred firft, the increafe of appetite or the *diuresis*; but fhe believes both about the fame time.

The medicines were ordered to be difcontinued; and a cordial mixture was prefcribed to be given three or four times a day, with a fuitable diet, exclufive of the ufual lactation.

May 12. The child continued to pafs an increafed quantity of urine only for a day or two after I laft faw him; fince which time the fwelling has returned to within half an inch of the girth it meafured on April 29; yet the appetite remains good, and his health in other refpects is apparently improving.

The powders and ointment to be repeated as at firft.

May

May 17. The ſwelling now meaſures half an inch more than when I firſt ſaw him. From the 12th to the 14th his mother inadvertently omitted giving him any medicine. He is now ſickly, has no appetite, nor any increaſe of urine; the breathing more troubleſome than ever.

Medicines to be continued.

May 22. The intumeſcence continues, but is not increaſed. Appetite ſtill bad; loſs of ſtrength and emaciation faſt returning.

The medicines to be yet continued.

June 5. No alteration of the ſwelling, but he gets weaker; draws but little milk from the breaſt, and takes no other food; waſtes in his fleſh, and the countenance is become pale and ſickly, with great torpor and inirritability of the whole ſyſtem. No ſenſible alteration from the medicines.

*Rep. pulv. cum duplo digit. et utatur ung. ut ante.*

10th. Symptoms ſtationary.

The powders to be repeated, increaſing the fox-glove to ten grains for twelve doſes; and the ointment to be continued.

17th. Every ſymptom the ſame as when I firſt ſaw him, except the ſwelling being ſomewhat larger. Evident danger of the integuments rupturing from their exceſſive diſtenſion, and not allowing of the ſmalleſt compreſſion. No probability, without ſome ſpeedy and effectual relief, that he can live many days.

The medicines failing to produce any ſenſible operation, were now ordered to be omitted; and the *paracentesis abdominis* was recommended. But the parents of the child objecting to have an operation performed, it was at preſent omitted; nor would they conſent to have any other remedy uſed, imagining it was impoſſible the child could live long, and that no operation or medicine would be likely to be of any uſe. Yet on the 21ſt in the evening, the father of the child came to requeſt I would perform the operation as a laſt reſource, becauſe I had ſaid that it might ſave the child's life; and he now thought that without immediate relief it could not poſſibly ſurvive in its preſent condition many hours. An unavoidable engagement prevented my attendance until ten o'clock the next morning, (June 22) when the ſtate of the child was ſuch as left me but little room to ſuppoſe he could live one hour; indeed, it had much the appearance of being then actually dying; for, together with a coldneſs of the whole ſurface, manifeſtly greater on the extreme parts of the body (though laying on its mother's lap in a cloſe room near to a fire) and a very weak ſlow pulſe, his eyes were fixed and half cloſed; and frothy mucus was, and had been for ſome hours, conſtantly iſſuing from his mouth and nose; the breathing short, tight, and intermitting; his countenance the most complete *facies hippocratica* I ever witnessed. He had not eaten any solid food for some days, entirely subsisting on lactation; nor of this could he take much, as the obtaining of it extremely distressed him, from the great difficulty with which he breathed.

The *paracentesis* with a small trocar was performed, by thrusting the instrument about one inch into the abdomen, on the left side a little below the *umbilicus*; which was done while the child was laying on the lap of its mother. On withdrawing the perforator, there forcibly burst from the canula a vast quantity of air, (I should think not less, and probably more than two gallons) with a hissing noise that was distinctly heard at many yards distance; immediately followed by a flow, at first in a full stream, afterwards more gradually, of a very foetid liquid, exactly similar to a *liquid fæcal stool*, amounting in the whole to about a pint and a half, and not varying in its appearance from the first to the last drop. In this liquid were a vast many lumps of undissolved *fæces* of various sizes, some smaller than a split pea, and some as large as could possibly pass through the canula, the internal diameter of which was exactly one sixth of an inch. A subsidence of the abdomen on the flowing out of the air was instantly visible; and in less than two minutes there was a most complete collapse, not any of the swelling remaining. A plaster with lint was put to the wound, (from which not a drop of blood issued) and a flannel rather moistened with spirits applied round the body. After the child had recovered the fright, and doubtless some pain occasioned by the puncture, which he did in one minute, it was a pleasing sight to see him turn and as it were solicit the breast of his mother, which he seized eagerly, and seemed to enjoy with peculiar satisfaction; at one moment leaving the nipple to look around, and then returning to it with increased pleasure, which he repeatedly did for a considerable time. Indeed his breathing and altered countenance (*vita quam a morte restaurata*) evidently shewed the ease and comfort he now enjoyed. He also swallowed a little warmed wine and water; and this was ordered to be repeated during the day, as a cordial, and to obviate fainting.

June 23. He has appeared to be very comfortable ever since the operation; breathes easier, is warmer, and in many respects better; but is extremely weak, and no part of the countenance except the eyes appear mended. He takes the breast freely. This evening the roller being somewhat loose, it was re-applied by his mother, who thought there was somewhat more swelling than when it was first put on. Ordered the following medicine.

℞. Tinct.

℞. *Tinct. cinchon.* ℥vj. *Sp. æth. vitr.* ʒj. *Syr. sim.* ℥ij. *Conf. arom.* ʒj. *Fol. digit. pulv. gr. ij. m. detur cochl. min. ter die.*

24th. Continues nearly in the fame fituation as yefterday, except that the abdomen is becoming hard and evidently increafing in fize. Makes water freely; but has not had any ftool fince the morning of the 22d, before he was tapped.

Ordered to perfift with the mixture; and to have in the evening a glyfter of fugared milk and oil, if no ftool fhould be difcharged before that time.

26th. He had a natural ftool, therefore the glyfter was omitted. He is now exceedingly improved in his health, takes the breaft frequently and eats food very often, makes urine in full quantity, is playful and apparently eafy, though the abdomen is again fwelled as high as the *scrobiculus cordis*, and meafures twenty-four inches and a half; tenfe, elaftic, and fonorous, when tapped with the hands. Has not had any ftool fince the evening of the 24th.

*Cap. illico jalap. gr. iv. Calom. gr. j. pro cathartico; dein persistat usu et ante mist. et ung. repet.*

27th. He had two or three ftools from the powder. Girth of the fwelling to day twenty-five inches, in other refpects full as well as yefterday; eats heartily, &c.

July 1. The fwelling continues ftationary, has natural ftools and they are of a thicker confiftence than ufual; voids much wind by the anus, makes water plentifully, appetite good.

The following was now prefcribed to ftrengthen the relaxed bowels, and to enable them to expel the air more freely.

℞. *Ext. cinchon.* ℈iv. *solv. ex. Aq. calid.* ℥ij. *Tinct. cinch.* ℥iſs. *Tinct. fer. mur. gt.* 80, *Syr. tolu.* ℥ivſs. *m. Detur.* ℥ſs. *ter die.*

9th. The fwelling is ftill twenty-four inches and a half over the abdomen, but it does not increafe upwards; continues in other refpects healthy; evacuates wind largely *per anum.*

*Cap. calom. gr. j. mane quartis diebus pro cathartico; et persistat mistura intermediis diebus.*

17th. The calomel gave him three or four ftools, and the fulnefs appeared afterwards to be fomewhat diminifhed, but is now of the fame girth as on the 9th, and appears to be extended more upwards. The whole of the fwelling feems to be palpably flatulent. In other refpects there is no change.

I now wifhed the paracentefis to be repeated, but his friends would not confent to it. The tonic mixture was therefore ordered to be continued, a carminative cathartic to be taken every fourth day, and the body to be immerfed in cold water every morning, except on the days of taking the purges.

Aug. 5th. He has been weaned the laſt four or five days; continues hearty, but with little alteration in the ſwelling. Has of late ſometimes had ſpontaneous *diarrhœa*, on conſideration of which the purges have not been adminiſtered; nor has he been bathed more than twice, on account of the trouble it gave to the aſſiſtants, and the fright it occaſioned to himſelf.

He was ordered to take ten drops of muriated tincture of iron three times a day.

*Sequel.*

What quantity he took of the muriated iron is uncertain, but I believe it was not much; nor after this time was any medicine given to him, except a purge occaſionally on account of obſtipation. In this interval another mode of evacuating the air, which was by introducing a glyſter-pipe into the rectum, was tried a few times on the ſuggeſtion of Dr. Darwin, (*Zoonom. vol.* 2); but it did not from any of the trials appear to have been uſeful, and was certainly leſs ſo than the exhibition of a common glyſter; and this, indeed, was generally neceſſary before the introduction of the pipe, as otherwiſe the end of it became ſtrongly clogged with hardened fæces. His friends would not conſent, as has been before ſtated, to have the paracenteſis repeated; ſo that it remains unknown what might have been the conſequence of reiterated perforations as a temporary or laſting relief. The diſeaſe, at length, occaſioned the death of the patient though he lived nearly eighteen months from the time of the operation. It ſomehow unfortunately happened that I did not hear of the fatal event until it was too late to examine the dead body; a circumſtance that I much regret, as diſſection (if it could have been obtained) might have clearly elucidated the real nature of the diſeaſe.

*Remarks on the above Caſe.*

The mode of treatment adopted in the preceding caſe, I think, clearly proves that ſome portion of water was contained in the abdomen along with the air; and that the mercury and fox-glove were the agents by which that fluid was expelled.

It may be inquired, ſince the intumeſcence was ſo conſiderably leſſened on the 5th of May, why I directed the medicines to be diſcontinued, and another plan to be proſecuted? Why there ſhould be any alteration propoſed, while the ſwelling was ſo kindly ſubſiding? To ſuch queſtions I can only reply by explaining the circumſtances that influenced my opinion at the time, which were, that from the very conſiderable diuretic operation of the medicines I had ſtrong reaſon to ſuſpect ſubſequent debility, debility, with naufea, and other effects that are well known to follow the exhibition of the diuretic I had made choice of, which fymptoms I wifhed to avoid; and from the ftate of the abdomen, I was well fatisfied that the water it contained was in trifling quantity; and confequently, that the remaining fwelling was chiefly, or entirely, flatulent. But to put this point entirely out of doubt, the *digitalis* was afterwards repeated, and its dofe increafed, until it appeared beyond all fufpicion that its further exhibition would be ufelefs.

The imminent danger in which the child appeared at the time the operation was performed, was fuch as to leave fcarcely any hope that he could have furvived it; yet, it is moft evident that without fuch relief as the perforation effected, he muft inevitably have died in a very fhort time.

I find, however, after a pretty extenfive fearch for the purpofe, that there are but very few inftances of the tympanites recorded (I have not met with any diftinctly related) which have been treated by the paracentefis, or puncture of the abdomen. But as in this cafe the operation was attended with fuch happy effect at the time, it is much to be regretted that I had not permiffion to repeat it as occafions might have required; by which, probably, the child's life might at leaft have been prolonged, though the difeafe might not have been cured.

The fæcal difcharge that followed the puncture muft have come from the bowels; but whether from perforation made by the inftrument in the operation, or from fome preternatural or originally formed aperture in the bowels coexiftent with the formation of the child, or from an inteftinal orifice any way occafioned by worms, or by difeafe, I cannot pretend to determine; and whether therefore this difeafe were originally and exclufively an abdominal, or an inteftinal cafe, perhaps cannot be very certainly pronounced; we only know, that whatever it were before the operation, from that time it muft have been of the latter kind, or a complication of each, confifting of air collected both within the bowels, and alfo in the peritonæal cavity. I think it not improbable, but in the original conformation of the inteftines, fome opening or aperture might have exifted; or worms in the inteftines might have produced fuch a perforation as would caufe the communication which probably occurred in the early part of the difeafe, or inflammation of the bowels, ending in a very limited fphacelation of a part of them, might and perhaps would occafion neceffarily a tympanitic affection. For it is difficult to conceive, how air can be generated in the cavity of the peritonæum in fuch prodigious quantity without fome communication with the inteftines; and it is very properly queftioned by a writer of fterling eminence, (Dr. Cullen) whofe whose opinion I shall quote below, whether such a case as the *T. abdominalis*, ever does exist without such communication?

The other remedies made use of, as they were of no material significance, it is unnecessary to comment upon.

### *A Critical Analysis of the Opinions of the principal Authors who have written on Tympanites.*

Most of the writers whose sentiments have been detailed on the subject of tympany, consider it as a disease of very rare occurrence, under whatever form it may appear; and there are some, it is to be suspected, as will be evident from the following analysis, who have detailed their opinions, while they have not really ever witnessed any case in support of them.

Celsus, a physician or surgeon, who practised early in the first century, has appropriated a part of one of his chapters "*de medicina*," to the cure of this disease. Indeed, his practical directions are most excellent, and clearly shew that he had seen the disorder he treats of. Vomiting, warm and dry fomentations, cupping and scarifying, and, as a last resource, *per alvum infundere aquam alidam*, are the remedies he recommends. He also advises vehement rubbing of the part three or four times a day with oil impregnated with some warm substance, such as mustard, *donec cutim erodat*; and the actual cautery to be applied to several parts of the belly, to produce exulceration; the ulcers to be kept a long time open. Boiled squill, he says, is also useful to be bound upon the skin. Lastly, he enjoins for a long time after the disease, *(posthac inflationes)* that the sick abstain from every thing of a flatulent nature. (*Celsus*, *lib.* 3, *cap.* 21.)

Ambrose Parey, whose works were made public more than two hundred years ago, (his epistle dedicatory bears date Feb. 1579) in the 11th chapter of his 8th book, treats of the dropsy. He describes the Tympanites, and what he calls the moist *ascites*; also particular dropsies, such as the *hydrocephalus*, *pleurocele*, *hydrocele*, &c. But it is remarkable, that notwithstanding he is very particular in his mode of curing the dropsy, in which he recommends bags, baths, liniments, emplasters, &c. and takes great pains to refute the mistaken opinion of Eristratus, who condemned the paracentesis as deadly; yet he does not give one word of direction respecting the treatment of the tympany; from which it may be very reasonably inferred, although his practice had been very extensive, having, as he says, enjoyed the office of principal chirurgeon under three kings, that he had really never seen the disease.

Van Swieten, in his Commentaries upon Boerhaave's Aphorisms, has collected much information from various authors respecting this disease. From the valuable and critical account account which he has given, there can be no doubt of the exiſtence of it, in the two forms which modern Noſologiſts have deſcribed. He has adduced inſtances of both; but one of the caſes (from the Med. Eſſays, Vol. I.) was evidently an inſtance of the *T. inteſtinalis*, which is probably the only ſpecies that is curable. The caſe which he mentions from *Helmont*, in which the ſubſidence of the abdomen was inſtantaneous after the operation of the paracenteſis, proved almoſt ſuddenly fatal from the operation; but why fatal is not accounted for. May it not have been from arterial hæmorrhage, as in wounding the epigaſtric artery with the inſtrument? Some inſtances of which we know to have happened, and are recorded in Medical Communications, Vol. II. This patient of Helmont's, it ſhould be noted, was tapped for an aſcites, nor was it known till after the operation what was the nature of the caſe.—Van Swieten's opinions of the paracenteſis in the tympany is, that the attempt would be hazardous; though he admits that where certain deſtruction is at hand, it is juſtifiable to uſe a doubtful remedy. He judiciouſly recommends that the trocar ſhould be of a ſmall ſize, that if a wound be inflicted in the inteſtine it may more certainly and ſpeedily cloſe, than if a large inſtrument were employed. In one of his obſervations he is unqueſtionably wrong; it is this, "In a tympany the abdomen is never diſtended to ſo vaſt a ſize as in an aſcites; but is flatter and more compreſſed towards the ſides, and more prominent before." (*Edin. edit. translated from the Latin, vol.* xii, *ſect.* 1226.) In the caſe above recorded, and in the only one other that I ever ſaw, the diſtenſion of the whole of the integuments of the abdomen was as great as could poſſibly be, and certainly in every part more tenſe than in any inſtance of aſcites.

Hoffman ſays, this is a diſtemper that rarely happens, and is of opinion that it ſhould rather be pronounced a ſymptom of the *anasarca* and *ascites*, than denominated a certain ſpecies of the dropſy. He recommends for the cure of it carminative glyſters; and affirms, that purging pills joined with an opiate have done great ſervice in this caſe; he alſo adviſes the whole of the abdomen to be anointed with camphorated oil for ſome days running, which gives ſome relief. (*Swan's edit. of Sydenham, 4th edit. p.* 514)

Juncker's opinion is, that powerful evacuations are not ſo uſeful in this diſeaſe as in the aſcites; but gentle evacuations by ſtool, urine and ſweat are beneficial. (*Ibid.*)

Heister ſays, in a tympany which is owing to watery humours, the relaxed tone of the viſcera, and wind, which greatly diſtend the flaccid bowels and abdomen, ſtrengthening and carminative medicines ſhould be mixed with reſolvents, bitters bitters and faline medicines, or both given alternately. He alfo recommends purging, a proper diet, and warm liniments or plafters for the abdomen. (*Ibid.*) The *T. abdominalis*, he afferts, is a cafe very rarely found, though an inftance of it quoted by him from RUYSCH, who performed the diffection in the prefence of Heifter, clearly evinces its exiftence. Heifter adds, " Dr. R. though a very old anatomift, had never feen an inftance of it before; and I, who am now feventy years of age, and have fince that time opened the bodies of many perfons fuppofed to have had a true tympanites, never afterwards met with any air in the cavity of the abdomen." (*Wirgman's translation of Heister's Med. Chir. et Anat. Cases*, 4*to p.* 21.)

Dr. MEAD, though fhort in his account of this difeafe, is very explicit. He diftinguifhes two kinds of tympany: one he calls abdominal, which is, when the abdomen is bloated up with a vapour, caufed by exhalation from fome mortified vifcus, of which he gives a notable inftance; fecond, the tympany which happens without any putrefaction, from an elaftic air engendered in the inteftines, which it ftretches to fuch a pitch as to deftroy their contractile power, &c. The firft fpecies, which proceeds from mortification, he believes to be abfolutely incurable. The other fpecies, he fays, is to be treated with moderate cathartics frequently adminiftered, and carminatives; with diet of very eafy digeftion. He alfo recommends exercife, and large glyfters of warm water, and cauterizing the abdomen as prefcribed by Celfus; or, in place of ufing the red hot irons, to lay blifters on the abdomen. (*Mead's Medical Precepts and Cautions*, *ch.* 8*th.*)

Sir JOHN PRINGLE (*Difeases of the Army*, 4to. edit. 5th. p. 214) had inftances of this difeafe, though, he fays, they " did not often occur;" he attributed them to " the unfeafonable ufe of opiates in the dyfentery, or of the bark in intermittents." The cafes he faw were diftenfions of the colon from air, and they yielded to rhabarbarine cathartics, aromatics, and tonics. " All ftrong phyfic, and carminatives without laxatives, were hurtful. If any fever was prefent he began the cure with bleeding, and gave the common faline mixture with rhubarb. From the event of one cafe, which ended fuddenly fatal, owing to the belly fubfiding all at once, the confequence of three or four loofe ftools, he advifes the patient to be fwathed, as thereby a compreffion can always be made fuitable to the decreafe of the air in the bowels.

CULLEN, the late learned Profeffor of the Practice of Phyfic at Edinburgh, has given a more full, accurate, and fcientific hiftory of this difeafe than any perfon before his time. He defcribes, in common with the moft correct of the Nofological writers, writers, two fpecies of the tympanites. 1. *T. inteftinalis.* 2. *T. abdominalis.* His idea of the proximate caufe of the difeafe is, that it confifts in "a lofs of tone in the mufcular fibres of the inteftines," and that the interruption of the paffage of the air therefrom is dependant upon "fpafmodic conftrictions in certain parts of the canal." This firft fpecies of the difeafe is the only one, in his opinion, that can be cured. The other fpecies, he thinks, is not a primary difeafe; is an affection of very rare occurrence, if indeed, as is very doubtful, it ever exifts feparately from the *inteftinalis*; is not eafily to be diftinguifhed; and when known, does not admit of a cure. Indeed, his opinion of the firft fpecies is, that it is generally a fatal difeafe, feldom curable, and hardly ever "but in a recent cafe." For evacuating the air accumulated in the inteftines he advifes gentle laxatives only," as ftrong ones, "in the tenfe and over-diftended ftate of the inteftines, would endanger the bringing on of inflammation." Glyfters alfo, when the fæces are in a "hard and dry ftate," become neceffary; and particularly when they produce "a confiderable evacuation of air; as they muft thereby have fome effect in relaxing the fpafms of the inteftines, they ought to be frequently repeated." For taking off the conftrictions of the inteftines, antifpafmodics of the milder kinds are recommended, and particularly "opium after the operation of a purgative." Fomentations and *tepid* bathing, long continued, have alfo been found ufeful for the fame indication. To reftore the tone of the relaxed bowels, tonic remedies, fuch as "chalybeates, bitters, or the Peruvian bark, he thinks, are properly indicated;" and as there is "no tonic remedy more powerful than cold applied outwardly, and cold drink taken into the ftomach," fo thefe have been conftantly prefcribed, and cold bathing has been advantageoufly ufed; but the moft efficacious application of cold feems to be that of applying "fnow to the lower belly, from which feveral inftances have occurred of the difeafe being fuddenly and entirely cured."* "Fofil acids and neutral falts, as antizymics, may be ufeful. As to the *paracentefis*, "in obftinate and defperate cafes, he fays, it has been propofed, but it is a very doubtful remedy, and there is *hardly any teftimony of its having been practifed with fuccefs*." He believes "this operation is a remedy fuited efpecially, and almoft only, to the *T. abdominalis*, which, however, is not very likely to be cured by this remedy; and how far the operation may be *fafe* in the *T.*

* Quere, Where are thefe Cafes recorded?

*inteſtinalis*, he thinks, has not been determined by any proper experience." (*Cullen's Firſt Lines*, *vol.* 4.)

Mr. Benjamin Bell, in his late Syſtem of Surgery, has communicated ſome valuable pathological information on this ſubject. Though that ſpecies of the diſeaſe which has the air confined in the inteſtines only is the moſt common, and which, he ſuppoſes, proceeds from the bowels loſing their tone entirely; yet he ſpeaks of one inſtance of the other ſpecies (or rather a mixture of the two) which fell under his own inſpection, and mentions another that he had heard of; in both of which the air was found to have eſcaped from the inteſtines by a "*very ſmall hole*" which was diſcovered in one of them.* He therefore thinks that the *T. abdominalis* very rarely proceeds from any other cauſe; and if it does, that no remedy will ever be able to effect a cure. However, from whatever cauſe the diſeaſe may have originated, and whether the air be contained in the bowels themſelves, or diffuſed in the cavity of the peritonæum; ſo ſoon as it is found to be productive of much diſtreſs, he thinks no doubt ſhould occur of the propriety of diſcharging it. Making a perforation into the abdomen for diſcharging air collected in the inteſtines, he obſerves, is no doubt a very formidable operation, and ought not to be attempted but in caſes of real neceſſity; but as death has often enſued from this diſeaſe, he is clearly of opinion, when all the uſual remedies have failed, that the operation ought to be performed rather than the patient ſhould be conſigned to die in certain miſery. He recommends uſing a trocar of the ſmalleſt ſize; but it does not appear that he ever performed the operation in this diſeaſe. (*Bell's Surgery*, *vol.* 2.)

Dr. Darwin (*Zoonomia*, *vol.* 2, *p.* 131.) alſo diſtinguiſhes two varieties of this diſeaſe. He ſays, that the air in the cavity of the abdomen is ſometimes in a few days exchanged for water, and the tympany becomes an aſcites.——Excluſive of ſome other remedies of uſual recommendation, he adviſes "the introduction of a glyſter-pipe into the rectum, to take off the reſiſtance of the ſphincter, and thus diſcharge the air." Improving on this idea, it has occurred to me that it would be practicable in this diſeaſe, having firſt emptied of their fæcal contents

* It would have been more ſatisfactory if Mr. B. had mentioned in what part of the inteſtines the holes were diſcovered, and whether in the ſame part in both patients. It may fairly be preſumed that there were no worms in the bowels in theſe caſes, or within the cavity of the peritonæum, otherwiſe ſuch an occurrnce could not fail to have been mentioned. Van Swieten (*Com. on Boerhaave*, *Sect.* 1226) has obſerved, if worms have bored through the inteſtines, an abdominal tympanites may take place.

contents the rectum and fuch part of the colon as an injection would reach, to *pump out air from the inteftines with fome mechanical contrivance*, fuch, for inftance, as was a few years ago invented by Mr. *Trye*, of Gloucefter, to extract urine from the bladder.* I confefs, the idea did not occur to me at the proper time, or I think I fhould have tried the operation of this ingenious contrivance. The introduction of a pipe or tube formed of a fufficient length and diameter, would hardly give more inconvenience to the patient, or trouble to the operator, than the paffing up of a common glyfter-pipe. The elaftic gum or *caoutchouc* is a fubftance admirably well adapted for the purpofe; and it might be made of fuch a length as to be introduced, I fhould conceive, without any hazard, through the whole length of the rectum into the colon itfelf; by which its power of extracting the air would be infinitely increafed. It fhould be well oiled at the time of its introduction; and if any rifk fhould arife of its collapfing from the warmth of the part, it might be conveniently kept permeable by having a fpirally formed wire contained within it.

Dr. Bailie, who is the laft writer I fhall mention on the fubject, imagines it is "very probable that in cafes of Tympanites the air is fecreted by the fmall veffels opening upon the villi of the internal membrane of the inteftines, and thrown into their cavity." (*Med. Chir. Tranf. vol.* 1, *p.* 207.) Of what kind thefe air-veffels are I have no adequate conception; but can readily fuppofe, that if there exift any veffels within the inteftines, or opening upon their internal furface, capable of fuch an operation as generating and expelling air, that the fame alfo may exift in the peritonæal cavity, and thus readily produce a tympanites. They may, indeed, as well be fuppofed to have exiftence in various other fituations or cavities of the body, and to give occafion to flatulent difeafes in thofe parts. But as the former propofition is, I believe, without proof, fo the latter muft be confidered (unfortunately for theory) as an affertion entirely gratuitous.

### *Of Acupuncturation.*

Quere.—In the peritonæal Tympany could occafional benefit be derived by acupuncturation of the abdomen? Being a more fimple operation than the paracentefis, it would of courfe have preference, at leaft with timid patients; but it is from practice only that we can learn whether it would be equally ufeful. This operation, I believe, never was practifed much, if

* See Trye's Remarks on morbid Retentions of Urine, 8vo. 1785.

 at at all, in this country, as it has been in fome parts of America;* and by the Chinefe and Japanefe it has always been an operation of practice, in a great number of diforders to which thofe people are liable, but particularly in a difeafe very analagous to the tympany. It is by the operation of the acupuncture that they cure a fpecies of cholic called *Senki*, which is faid to proceed from a rarefaction of fome pernicious vapour in the cavity of the abdomen. Heifter (*General Syftem of Surgery, vol.* 1, *part* 2, *ch.* 18.) very fuperficially mentions this operation. He has given drawings of two inftruments which the Japanefe make ufe of in performing it; but obferves, that the practice has not been followed by any of our European nations; and therefore directs his readers for further information to confult the writings of RHYN and KEMPFER, two furgeons who had been fpectators of the operation in that country. But the beft account I have feen of the acupuncture occurs in the ninth vol. of "*The modern part of an Univerfal Hiftory, from the earlieft account of times*, 8*vo*." As the frequency of the operation demonftrates at leaft its fafety, it may yet perhaps be thought by the Englifh Surgeons on fome occafions to be worth imitating; and as the method of doing it is both curious and but little known, the following detail of it, from the volume mentioned, is tranfcribed for more public information, and to conclude the fubject: "The place made choice of for the puncture is commonly at a middle diftance between the navel and the pit of the ftomach; but often as much nearer to or farther from either, as the operator, after a due fcrutiny, thinks moft proper; and in this, and the judging rightly how deep the needle muft be thruft below the fkin, fo as to reach the feat of the morbific matter, and giving it a proper vent, confifts the main fkill of the artift, and the fuccefs of the operation is faid to depend. Each row hath its particular name, which carries with it a kind of direction with regard to the depth of each puncture, and the diftance of the holes from each other; which laft feldom exceeds half an inch in grown perfons in the perpendicular rows, though fomething more in thofe which are made acrofs the body, thus,

. . .
. . .
. . .

"The needles which perform the operation are made, as was hinted at firft, either of the fineft gold or filver, and without the leaft drofs or alloy. They muft be exquifitely flender, finely polifhed, and carry a curious point, and with fome degree

* See Dampier's Voyages.

gree of hardnefs, which is given them by the maker by tempering, and not by any mixture, in order to facilitate their entrance, and penetrating the fkin. But, though the country abounds with expert artifts, able to make them in the higheft perfection, yet none are allowed to vend them but fuch as are licenfed by the Emperor.

"These needles are of two forts with refpect to their ftructure, as well as materials; the one, either of gold or filver indifferently, and about four inches long, very flender, and ending in a fharp point, and have at the other end a fmall twifted handle, which ferves to turn them round with the extremity of the middle finger and thumb, in order to fink them into the flefh with greater eafe and fafety; the other fort is chiefly of filver, and much like the firft in length and fhape, but exceedingly fmall towards the point, with a fhort thick handle, chanelled for the fame end of turning them about, and to prevent their going in too deep; and, for the fame reafon, fome of them are cafed in a kind of copper tube, of the bignefs of a goofequill, which ferves as a fort of gauge, and lets the point in juft fo far as the operator hath determined it. The beft fort of needles are carefully kept in a cafe made of bull's horn, lined with fome foft downy ftuff. This cafe is fhaped fomewhat like a hammer, having on the ftriking fide a piece of lead, to give it a fufficient weight, and on the outfide a compreffed round piece of leather, to prevent a recoil, and with this they ftrike the needle through the thicknefs of the fkin; after which, they keep turning the handle about with the hand till it is funk to the depth they defign it, that is, till it is thought to have reachad the feat of the morbific virus, which, in grown perfons, is feldom lefs than half, or more than a whole inch. This done, he draws it out, and compreffes the part, in order to force the morbific vapour or fpirit out. The directions and nice rules for the performing of this curious operation are many, and require great fkill and attention in the operator; and, when duly performed, may be of excellent ufe not only againft the excruciating diftemper called Senki, but againft many other topical ones, which are more commonly cured by the Indian *Moxa*, and other cauftics. On the other hand, thefe laft are often tried againft the diftemper above-mentioned, by applying the cauftic to the belly, on each fide of the navel, and about two inches from it, but moftly without any fuccefs, it being very unlikely that fuch an application fhould reach the feat of the diftemper; whereas the benefit which hath accrued from the acupuncture, in that one difeafe, hath encouraged others to apply it indifferently to other parts of the body, where the *Moxa* is ufed; and, by a due care and precaution not to prick any nerves, nerves, tendons, or other confiderable blood-veffels, have cured their patients by it, without putting them to the excruciating torture which attends that of the *Moxa*, or other cauftics.

